# Phenotypic immune characterization of gastric and esophageal adenocarcinomas reveals profound immune suppression in esophageal tumor locations

**DOI:** 10.3389/fimmu.2024.1372272

**Published:** 2024-04-04

**Authors:** Tessa S. Groen-van Schooten, Micaela Harrasser, Jens Seidel, Emma N. Bos, Tania Fleitas, Monique van Mourik, Roos E. Pouw, Ruben S. A. Goedegebuure, Benthe H. Doeve, Jasper Sanders, Joris Bos, Mark I. van Berge Henegouwen, Victor L. J. L. Thijssen, Nicole C. T. van Grieken, Hanneke W. M. van Laarhoven, Tanja D. de Gruijl, Sarah Derks

**Affiliations:** ^1^ Department of Medical Oncology, Amsterdam University Medical Center (UMC) location Vrije Universiteit Amsterdam, Amsterdam, Netherlands; ^2^ Cancer Biology and Immunology, Cancer Center Amsterdam, Amsterdam, Netherlands; ^3^ Oncode Institute, Utrecht, Netherlands; ^4^ Medical Oncology Department, Instituto Investigación Sanitaria INCLIVA (INCLIVA), Hospital Clínico Universitario de Valencia, Universitat de Valencia, Valencia, Spain; ^5^ Department of Gastroenterology, Amsterdam University Medical Center (UMC) location Vrije Universiteit Amsterdam, Amsterdam, Netherlands; ^6^ Department of Surgery, Amsterdam University Medical Center (UMC), University of Amsterdam, Amsterdam, Netherlands; ^7^ Amsterdam University Medical Center (UMC) location Vrije Universiteit Amsterdam, Radiation Oncology, Amsterdam, Netherlands; ^8^ Laboratory for Experimental Oncology and Radiobiology, Center for Experimental and Molecular Medicine, Amsterdam, Netherlands; ^9^ Department of Pathology, Amsterdam UMC location Vrije Universiteit Amsterdam, Amsterdam, Netherlands; ^10^ Imaging and Biomarkers, Cancer Center Amsterdam, Amsterdam, Netherlands; ^11^ Department of Medical Oncology, Amsterdam University Medical Center (UMC), University of Amsterdam, Amsterdam, Netherlands

**Keywords:** tumor microenvironment, single cell flow cytometry, biomarkers, HER2, MSI

## Abstract

**Background:**

Tumors in the distal esophagus (EAC), gastro-esophageal junction including cardia (GEJAC), and stomach (GAC) develop in close proximity and show strong similarities on a molecular and cellular level. However, recent clinical data showed that the effectiveness of chemo-immunotherapy is limited to a subset of GEAC patients and that EACs and GEJACs generally benefit less from checkpoint inhibition compared to GACs. As the composition of the tumor immune microenvironment drives response to (immuno)therapy we here performed a detailed immune analysis of a large series of GEACs to facilitate the development of a more individualized immunomodulatory strategy.

**Methods:**

Extensive immunophenotyping was performed by 14-color flow cytometry in a prospective study to detail the immune composition of untreated gastro-esophageal cancers (n=104) using fresh tumor biopsies of 35 EACs, 38 GEJACs and 31 GACs. The immune cell composition of GEACs was characterized and correlated with clinicopathologic features such as tumor location, MSI and HER2 status. The spatial immune architecture of a subset of tumors (n=30) was evaluated using multiplex immunohistochemistry (mIHC) which allowed us to determine the tumor infiltration status of CD3+, CD8+, FoxP3+, CD163+ and Ki67+ cells.

**Results:**

Immunophenotyping revealed that the tumor immune microenvironment of GEACs is heterogeneous and that immune suppressive cell populations such as monocytic myeloid-derived suppressor cells (mMDSC) are more abundant in EACs compared to GACs (p<0.001). In contrast, GACs indicated a proinflammatory microenvironment with elevated frequencies of proliferating (Ki67+) CD4 Th cells (p<0.001), Ki67+ CD8 T cells (p=0.002), and CD8 effector memory-T cells (p=0.024). Differences between EACs and GACs were confirmed by mIHC analyses showing lower densities of tumor- and stroma-infiltrating Ki67+ CD8 T cells in EAC compared to GAC (both p=0.021).

**Discussions:**

This comprehensive immune phenotype study of a large series of untreated GEACs, identified that tumors with an esophageal tumor location have more immune suppressive features compared to tumors in the gastro-esophageal junction or stomach which might explain the location-specific responses to checkpoint inhibitors in this disease. These findings provide an important rationale for stratification according to tumor location in clinical studies and the development of location-dependent immunomodulatory treatment approaches.

## Introduction

Gastroesophageal adenocarcinomas (GEACs) are among the deadliest malignancies, with more than 1.6 million new cases and over 1.3 million new deaths in 2020 worldwide (1). GEACs comprise tumors located in the esophagus (EAC), in the gastroesophageal junction (GEJAC) and in the stomach (GAC). Although these cancers develop in close proximity, curative treatment plans depend on the tumor location. Locally advanced esophageal cancers are often treated with neoadjuvant chemoradiotherapy followed by a tumor resection and adjuvant immunotherapy (2, 3), while gastric adenocarcinomas are treated with a tumor resection and perioperative chemotherapy including a fluoropyrimidine, a platinum compound and docetaxel (FLOT) (4, 5). For GEJ tumors, both treatment plans are optional. In the metastatic setting, adenocarcinomas of the esophagus and stomach are treated with the same treatment schedule and most often with a platinum and fluoropyrimidine containing doublet chemotherapy combined with trastuzumab or nivolumab depending on expression of Human Epidemal growth factor Receptor 2 (HER2) expression and the combined positive score for expression of Programmed death-ligand 1 (PD-L1) (6, 7).

In clinical practice, it is often difficult to determine the exact tumor location. The Siewert classification is used to subcategorize GEJACs based on the anatomical distance between the junction and the tumor core (8). Siewert type I cancers originate from the esophagus (1–5 cm above the junction) and are defined as EACs; Siewert type II cancers arise in the junction and can be located 1 cm above to 2 cm below the junction; Siewert type III refers to gastric adenocarcinomas located 2–5 cm below the junction. Also on a molecular level, EACs, GEJACs and GACs show much resemblance. Genome and transcriptome studies such as those from The Cancer Genome Atlas (TCGA) showed that while GACs are subdivided in 4 molecular subtypes, i.e. those with microsatellite instability (MSI), genome stability (GS), chromosomal instability (CIN) and lastly, Epstein-Barr virus (EBV) positivity (9), the vast majority of EACs and GEJACs belong to the CIN subgroup of cancers (10). Aberrant DNA methylation levels, however, were more often observed in CIN tumors located in the esophagus and GE junction compared to tumors in the stomach, indicating location-specificity within the molecular subgroups (10).

In a recent study we have shown that the molecularly distinct subtypes also differ in immune cell composition (11). While MSI+ and EBV+ GEAs contain a higher number of T cells, CIN GEAs are generally T cell excluded, although some inter-tumor heterogeneity was observed. In a separate study we showed that relatively higher levels of infiltrating T cells and a high intratumoral CD8:CD163 ratio in EACs predisposes for a complete pathological response to neoadjuvant chemoradiotherapy (nCRT) in EACs (12). Besides response to nCRT, the composition of the TME may also impact response to immunotherapy (13).

Interestingly, a recent subgroup analysis of the CheckMate 649 trial (14), showed that the survival benefit of Nivolumab addition to chemotherapy was mainly observed in patients with GAC (HR 0.76, 95% CI 0.66-0.87), and less in patients with GEJAC (HR 0.90, 95% CI 0.67-1.20) and EAC (HR 0.82, 95% CI 0.60-1.13). This difference in survival per primary tumor location was not observed in the control arm receiving chemotherapy alone. As the composition of the immune microenvironment impacts response to (immuno) therapy, we hypothesize that GEJACs and EACs have a more suppressed immune microenvironment compared to tumors in the stomach. To increase our understanding of the immune microenvironment of GEACs, we performed a detailed analysis of the immune features of a large series of GEACs (n=104) with different tumor locations and molecular features. We identified that GEACs with an esophageal tumor location had more immune suppressive features compared to adenocarcinomas in the stomach, which should be taken into account in designing future immunotargeting studies.

## Materials and methods

### Patient material

An observational prospective study to collect fresh tumor biopsies from EACs, GEJACs and GACs was performed between 2019 and 2021 at Amsterdam UMC. All patients gave informed consent for collection and immune analyses, according to institutional regulations. During pre-treatment endoscopy by an expert gastroenterologist, multiple tumor biopsies were collected for immediate processing or storage for later use (paraffin and snap frozen). Tumor locations were assessed as follows based on the bulk of the tumor: esophageal adenocarcinomas above the junction were annotated as EAC, gastro-esophageal junction tumors including cardia tumors were designated GEJAC and non-cardia gastric adenocarcinomas formed the GAC group. Biopsies fixed in formalin were paraffin embedded and stained with H&E for histological assessment, and annotated for representative tumor areas by an expert pathologist prior to processing for downstream applications. Clinicopathological characteristics, including age, sex, Lauren’s classification and clinical TNM stage, were recorded at the time of diagnosis. HER2 and MSI status were established following local hospital protocols for standard of care: HER2 positivity was assigned to tumors with immunohistochemistry (IHC) HER2 scores of 3+ or 2+ and HER2 *in situ* hybridization (ISH)+. MSI status was determined via IHC MMR status on MLH-1, MSH-2, MSH-6 and PMS-2. PD-L1 scoring and genome sequencing was not performed routinely in our center at the time of this study.

### Tumor dissociation and flow cytometry

Fresh tumor biopsies were collected in Dulbecco’s Modified Eagle Medium (DMEM) supplemented with 10% Fetal Calf Serum (FCS) on ice and immediately processed and stained for flow cytometry, (**Supplementary Table 1**) as described before (12). Data acquisition was performed on an LSR Fortessa flow cytometer (BD Biosciences, CA, USA). Flowjo™ Software version 10.2 for Windows was used to perform immune subtype analyses (**Supplementary Figures 1**, **2**).

### Multiplex IHC

Additional spatial immune profiling was performed with multiplex immunofluorescence staining in a subset of the tumors (*n* = 30) by a previously described method (10). In short, the OPAL 7-color fluorescence immunohistochemistry (IHC) kit (Akoya Biosciences, USA) was used following the manufacturer’s instructions to stain for human cytokeratin (anti-CK, clone AE1/AE3 (Dako)), CD8+ cells (Anti CD8 clone C8/144B (Dako)), CD3+ cells (anti-CD3 polyclonal (Dako)), FoxP3 + cells (anti-FoxP3 clone 236A/E7 (Abcam)), CD163+ cells (anti-CD163 clone 10D6 (Novocastra)) and Ki67+ cells (anti-Ki67 clone SP6 (Abcam)). Slides were stored at 4°C until imaging. Whole slide and multispectral imaging were done using the Vectra^®^ Polaris™ multispectral scanning microscope (Akoya Biosciences, USA). Multispectral images were unmixed and analyzed per tumor case in INFORM^®^ (Akoya Biosciences, USA). All data was exported for analysis with the phenoptrReports package (Akoya Biosciences, USA) in RStudio (RStudio, Inc., Boston, MA, USA).

### Statistical analysis

Statistical analyses were performed using R version 4.3.0 (R Core Team, 2022). Given the non-normality of the data, group differences were evaluated utilizing the non-parametric Dunn’s test via the dunn_test function from the rstatix package (version 0.7.2). The Vargha and Delaney A, a non-parametric effect size measure, was computed using the ‘muliVDA’ function of the rcompanion package (version 2.4.34). For visualization purposes, a pseudocount of 1 was added to each value of the multiplex IHC data before log-transformation. Importantly, statistical calculations were conducted on the non-transformed data. A p-value of ≤ 0.05 was considered statistically significant.

## Results

### Patient characteristics

During this prospective study, a total of 104 patients were included. The patient characteristics are presented in **Table 1**. In total, in 34% (35/104) of the patients the tumor was located in the distal esophagus (EAC), 36% (38/104) in the GEJAC, and 30% (31/104) of tumors were true GAC (16 located at the body/fundus and 15 at pylorus/antrum). The median age of all patients was 66 years (range 31-87 years of age). 68% (71/104) of all GEAC patients were male: for EAC 89% (31/35), for GEJAC 63% (24/38) and for GAC 52% (16/31). All EACs have an intestinal type tumor morphology while GEJAC and GACs additionally indicated a diffuse type morphology in 10.5% and 35.5% of cases. In this cohort, most EAC (97%, 34/35) were locally advanced adenocarcinomas (Stage III), while 24% of GEJAC (9/38) and 39% (12/31) of GAC had distant metastases. As EBV and MSI are rare in the esophagus, they are not routinely tested in EACs. EBV status was determined in 44 patients (18 GEJAC and 26 GAC) of which 3 GAC tumors were EBV positive. MMR deficiency was not detected in EACs (0/16) and occurred in 23.8% (5/21) GEJACs and 17% (4/23) GACs. HER2 positivity was detected in 21.2% (21/98) and more often present in EACs and GEJACs compared to GACs (25.7%, 21.1% and 12.9% respectively).

**Table 1:** Patient characteristics table.

	EAC		GEJAC		GAC		Total	
	N	%	N	%	N	%	N	%
**Patients enrolled**	35	34	38	37	31	30	104	100
**Age (median, range, Y)**	66	43-81	66	31-82	70	42-87	66	31-87
**Sex**								
Male	31	88.6	24	63.2	16	51.6	71	68.3
Female	4	11.4	14	36.8	15	48.4	33	31.7
**Lauren's classification**								
Intestinal	35	100.0	31	81.6	17	54.8	83	79.8
Diffuse	0	0.0	4	10.5	11	35.5	15	14.4
Mixed	0	0.0	3	7.9	2	6.5	5	4.8
Medullary	0	0.0	0	0.0	1	3.2	1	1.0
**cTNM staging**								
Primary tumor (T)								
1	0	0.0	0	0.0	0	0.0	0	0.0
2	4	11.4	6	15.8	1	3.2	11	10.6
3	30	85.7	24	63.2	20	64.5	74	71.2
4	1	2.9	4	10.5	7	22.6	12	11.5
x	0	0.0	1	2.6	3	9.7	4	3.8
Regional lymph nodes (N)								
0	10	28.6	9	23.7	6	19.4	25	24.0
1	12	34.3	11	28.9	10	32.3	33	31.7
2	11	31.4	12	31.6	11	35.5	34	32.7
3	2	5.7	6	15.8	4	12.9	12	11.5
Distant metastasis (M)								
0	34	97.1	27	71.1	19	61.3	80	76.9
1	1	2.9	9	23.7	12	38.7	22	21.2
**Molecular characterisation**								
MSI	0	0.0	5	19.2	4	14.8	9	8.7
MSS	16	100.0	21	80.8	23	85.2	60	57.7
EBV+	0	0.0	0	0.0	3	11.1	3	2.9
**HER2 status**								
Positive	9	28.1	8	22.9	4	12.9	21	20.2
Negative	23	71.9	27	77.1	27	87.1	77	74.0

Flow cytometry patient characteristics per tumor location. MSI, microsatelite instable; MSS, Microsatelite stable; EBV, Epstein Barr Virus.

### Phenotypic analysis reveals a more active T cell phenotype in GAC compared to EAC and GEJAC

**Figure 1:**
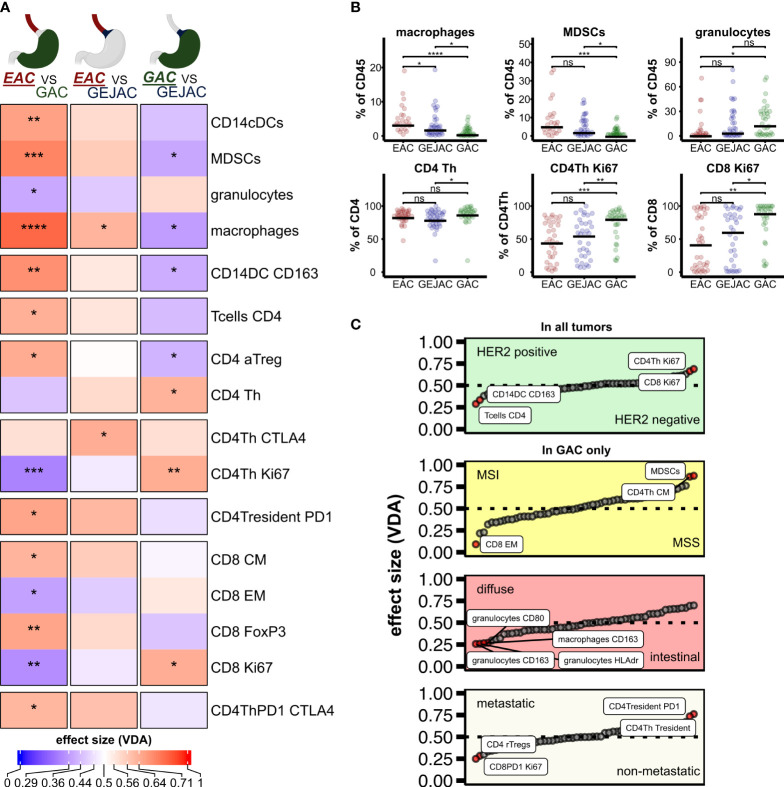
Differential analysis of immune subsets per tumor location. **(A)** Heatmap of Dunn estimates (average rank difference) between the 3 tumor locations. Reference group is underlined. Asterisks indicate p < 0.05. **(B)** Differential cell types between tumor locations, dunn-test, p adjusted for group comparison. **(C)** Main effects of clinicopathological characteristics. ns, not significant.

**Figure 2:**
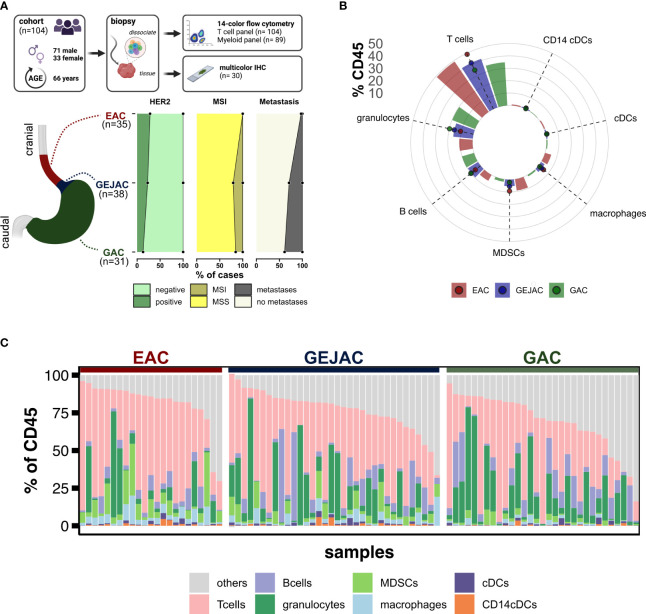
Study overview and distribution of main immune cells. **(A)** Methodology and samples. **(B)** Relative abundances of major immune cell types relative to CD45. **(C)** Relative immune cell abundances per sample.

Using two fresh tumor biopsies per tumor, we determined the presence of main immune cell subsets in a total of 104 tumors with flow cytometry (**Figure 1A**). Based on the number of viable cells per flow panel we were able to analyze T cell subsets in 104 tumors and myeloid subsets in 89 tumors. An overview of the cohort and methodology can be found in **Figure 2A**. For the gating strategy of the various immune subsets, we refer to **Supplementary Figures 1**, **2**.

We first compared the main immune cell populations relative to CD45 per tumor and identified T cells as the most abundant cell type in GEACs, followed by (CD15+CD11c+) granulocytes and (CD19+) B cells (**Figure 2B**) which was independent of tumor location. Next, we identified large heterogeneity in the immune cell composition of GEACs (**Figure 2C**). CD3+ T cell numbers (relative to CD45+) ranged from 0.3 to 85.9% (median 36.6%) which was also observed for granulocytes (range 0.0 – 81.0%, median 6.0%) and B cells (range 0.1 – 62%, median 4.6%). We next analyzed the association between tumor immune composition relative to CD45 and tumor location and identified substantial differences between EAC, GEJACs and GACs (**Figures 1A, B**; **Supplementary Table 2**). EACs had significantly higher mMDSC frequencies compared to GACs (6.1% vs 0.9%, p<0.001). GEJACs had a mMDSC frequency of 2.9% (vs 0.9% in GAC, p=0.02) which was right in between. The same pattern was observed for macrophages: EACs had a 4-fold higher frequency of macrophages compared to GAC (3.8% vs 0.9% p<0.001), while GEJAC had a 2-fold higher frequency (2.3% vs 0.9%, p=0.01) in macrophages compared to GAC. Granulocytes were enriched in GAC compared to EAC (14.8% vs 2.7%, p=0.042, **Figures 1A, B**).

We next explored lymphocyte activation and differentiation (the T cell panel; relative to CD3) in these cancers (**Supplementary Table 2**). Although the total number of CD3 T cells did not differ between locations, Ki67+ CD4+ Th cells were more abundant in GACs compared to GEJAC (83% vs 57.6%, p=0.005) and EAC (47.2%, p<0.001, **Figure 1B**). The same was observed for Ki67+ CD8 T cells, which were also more abundant in GACs (91.4%) compared to GEJAC (63.3%, p=0.01) and to EACs (44.4%, p=0.002, **Figure 1B**). CD8+ (CD27-45RA-) Effector Memory (EM) cells, on the other hand, were more abundant in EAC compared to GACs (p=0.024), indicating an enhanced memory response specifically in EAC.

### Differential histology, stage, MSI+ or HER2+ status does not explain the location specific immune infiltrate

We next determined whether enrichment of MSI status, diffuse type histology or metastatic status in GACs could account for differential immune subset content observed in the tumors in relation to esophageal-to-gastric location.

We first assessed immune infiltrate in relation to MSI status, and confirmed that compared to Microsatellite stable (MSS) cases, MSI GEACs have higher number of CD8 EM T cells (median frequency of 73.3% vs 51.5%, p=0.011), less CD4 Th central memory (CM) cells (median of 24.4% vs 44.4%, P=0.023), and less mMDSC (median 0.5% vs 1.8%, p=0.019, **Figure 1C**). We then evaluated the differences between tumor locations without MSI cases (**Supplementary Table 4**), and EACs still had significantly more mMDSCs and macrophages, and less proliferating Th cells and CD8+Ki67+ cells compared to GAC. We next assessed the influence of Lauren classification. While diffuse type GACs had higher frequencies of granulocytes compared to intestinal type GACs (**Figure 1C**; **Supplementary Table 5**), removing these cases did not change the differential immune analysis between tumor locations (**Supplementary Table 6**).

The same accounted for the influence of metastasis: although metastatic cancers harbored higher rates of Ki67+PD1+ CD8 T cells and Tregs (**Figure 1C**; **Supplementary Table 7**), excluding all stage IV cancers from location-immune analyses did not change our differential analysis results (**Supplementary Table 8**).

Although HER2 positive cancer was detected at all three tumor locations we analyzed the immune features of HER2 postive cancers as well and observed that HER2 positivity is associated with a significantly higher percentage of CD163+CD14+ conventional Dendritic cells (cDC) and CD4+ T cells, and less Ki67+ CD4 Th cells and Ki67+ CD8 cells compared to HER2 negative tumors (**Figure 1C**; **Supplementary Table 9**). For both HER2 negative as positive GEACs, EACs harbor more mMDSCs and macrophages compared to GACs (for both populations p<0.001, **Supplementary Tables 10**, **11**).

### Multiplex IHC confirms a more T cell inflamed tumor microenvironment in GACs compared to EACs

**Figure 3:**
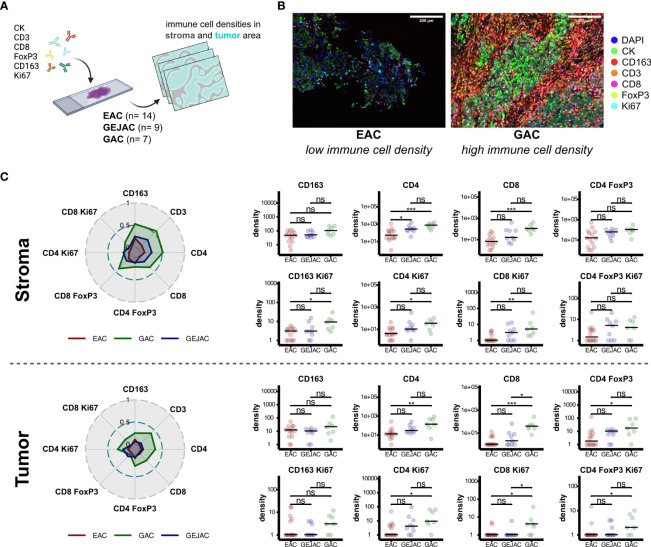
Multiplex immunohistochemistry reveals increased immune cell densities in gastric adenocarcinoma. **(A)** IHC method overview. **(B)** Representative images depicting increased CD4 and CD8 abundances in tumor locations. **(C)** Dominant immune cell subsets in stromal and tumor regions (left). Radar plots depict mean immune cell densities after minmax normalization (middle). Pairwise-comparisons of immune subsets between tumor locations (right). ns = not significant, * = p value < 0.05, ** = p value < 0.01, *** = p value < 0.001.

We next complemented our flow cytometry findings with multiplex immunohistochemistry (mIHC) on 30 GEACs (n=14 EAC; n*=*9 GEJAC; n=7 GAC (**Figures 3A, B**; **Supplementary Tables 12**, **13**) and confirmed that EACs had lower median densities of CD3+ cells in general (14.0 vs 167.0 cells/mm2 p=0.005) and both CD4 cells (11.5 vs 130.0 cells/mm2, p=0.006) and CD8 cells (0.0 vs 37.0, p<0.001) compared to GACs in tumor and even more significant in stroma (**Figure 3C**). Also, a higher median density of 3.0 and 4.0 (Ki67+) proliferating CD8 T cells/mm2 were found in GACs compared to EAC (p=0.021 and p=0.006) for both tumor and stroma respectively, which was in line with the immune phenotyping via flow cytometry. Again, immune cell densities of GEJAC were mostly in between EACs and GACs. In this analysis, 4 cases of MSI were included (n=3 for GEJAC; *n=*1 GAC). After excluding the MSI cases we still confirmed the lower CD4 and CD8 T cell densities in EAC compared to GAC. However, intratumoral proliferating T cells were not significantly lower in EAC than in GAC after removal of MSI cases (**Supplementary Table 14**).

## Discussion

To the best of our knowledge this is the first comprehensive phenotypic characterization of the immune infiltrate in a large series of gastric and esophageal adenocarcinomas on a single cell level. Using 14-color flow cytometry on fresh tumor biopsies of patients with adenocarcinomas in the esophagus, gastro-esophageal junction or stomach we identified that EACs have more suppressive immune cell populations compared to GEJAC and GACs. While the tumors show a high molecular resemblance, adenocarcinomas in the esophagus in general had a higher number of macrophages and mMDSCs compared to tumors in the GEJ or stomach, whereas cells associated with an active anti-tumor immune response such as proliferating CD4 Th cells or CD8 T cells were more abundant in the stomach compared to the esophagus. These differences were observed after correction for differences in disease stage, Lauren classification, MSI status and HER2 positivity.

This study complements previous genome and transcriptome studies which identified that esophageal adenocarcinomas show molecular resemblance with the CIN subtype of gastric cancer and can therefore be considered as virtually the same disease with a universal treatment approach. However, on an immunological level there are clear differences. The finding that EACs have more immune suppressive features accompanying higher T cell infiltration rates (thus allowing for immune escape) might explain why GEACs with a gastric tumor location benefit more from the addition of nivolumab to chemotherapy in the Checkmate 649 study as compared to patients with tumors located in the esophagus or GEJ. Together these findings indicate that EACs and GEACs can be considered as distinct entities and should be analyzed separately, at least in trials testing immunomodulatory strategies.

As EACs seem to benefit less from checkpoint inhibitors, these cancers likely need additional immunomodulation to overcome this resistance. A potential strategy is targeting M2 macrophages, for instance via inhibiting macrophage recruitment and proliferation with CSF-1R inhibitors. Several phase I and II studies have tested CSF-1R targeting drugs in the form of monoclonal antibodies or tyrosine kinase inhibitors to overcome the tumor resistance in macrophage-rich solid tumors. Although these drugs were considered safe, they did not show enough responses for continued testing (15, 16). An alternative strategy is to target the recruitment of macrophages by tumor cells through inhibiting the CCL2-CCR2 axis with CCL2-neutralizing antibodies which is currently being tested in metastatic castrate-resistant prostate cancer (NCT00992186). Unfortunately, so far none of these studies have included GEACs.

It is not immediately clear why esophageal adenocarcinomas have distinct immune features compared to gastric adenocarcinomas but what is known is that EACs develop in a background of chronic inflammation due to acid reflux. In the so-called Barrett’s esophagus an acute inflammatory state (esophagitis) evolves towards a state of chronic inflammation characterized by presence of IL-4 and IL-13, suppressive M2 macrophages and MDSCs (17–20). For adenocarcinomas in the stomach this is different as among other factors the gut microbiota shapes the tumor immune microenvironment and often have an immune promoting effect (21). Especially H. Pylori is an important inflammatory risk factor, as it is carried by around 60% of the world population and estimated to be responsible for 50% of GC cases (22). Although *H. pylori* is a noninvasive organism, it stimulates a robust inflammatory and immune response by the production of factors such as vacuolating cytotoxin A (VacA), cag pathogenicity island, cytotoxin-associated gene A (CagA), peptidoglycan outer membrane proteins, and γ-glutamyl transpeptidase (GGT) (23). Furthermore, other tumor location specific inflammatory risk factors that differ between EAC and GAC include infections with the Epstein Barr Virus, previous gastric surgery, pernicious anemia and auto-immune gastritis and may also cause dissimilarities in the immune repertoires (24).

At last, the general lower pH of the stomach prevents bacterial growth and infection but also effects immune function; i.e. an acidic pH is usually associated with suppression of immune effector function and upregulation of immune checkpoints such as TIM-3 LAG-3 and CTLA-4 (25). This is the opposite of what is observed in our study and does not explain the differences between immune compositions of cancer in the esophagus and stomach. Therefore, location specific drivers of the inflammatory state in EAC and GAC need further investigation, especially in relation to the tumor immune microenvironment and response to checkpoint inhibitors.

Besides studying location specific immunological characteristics of GEACs, we also determined the immunological characteristics of HER2 positive cancers. Comparing HER2 positive and HER2 negative cancers we identified that HER2 positivity is associated with enrichment of CD4 T cells and less proliferating CD4 Th cells and proliferating CD8 T cells compared to HER2 negative tumors. These findings are in agreement with studies in breast cancers that showed that HER2 positive breast cancers are characterized by a higher number of lymphocytes and tumor associated macrophages, compared to HER2 negative breast cancer (26). Also, for HER2 positive disease, tumors in the esophagus had more immune suppressive cell populations such as MDSCs and macrophages compared to tumors in the stomach which can potentially impact the additive effect of checkpoint inhibitors to chemo-trastuzumab in GEACs.

Interestingly, a subgroup analyses of the recently published KEYNOTE-811 study which demonstrated an additive effect of pembrolizumab to trastuzumab-chemotherapy (27), in treatment naïve advanced HER2 positive GEACs with a PD-L1 combined positive score (CPS)>1, showed that this effect was more pronounced in tumors with a location in the stomach (HR 0.7) compared to tumor in the GEJ (HR 0.85). Hopefully, translational studies connected to the KEYNOTE-811 give insight in tumor location specific anti-tumor immune responses.

One unexpected finding in our study is that we identified relatively high T cell proportions compared to our previous work using similar techniques (12). Although our previous studies did not include gastric cancers, the comparatively high T cell numbers might be explained by selective cell death of myeloid cells during tumor dissociation and staining procedures. Furthermore, we were not able to correlate immune profiles with response to therapy as EACs and GACs were treated differently in the non-metastatic setting. Further clinical studies are needed to determine how immune cell composition influences response to chemo- and immunotherapy.

In conclusion, we here identified that EACs differ markedly from GACs in terms of immune infiltrate, with the latter more Ki67+ CD8+ T cell inflamed and EAC with more macrophages and mMDSC content. This indicates that tumor location can be a defining feature influencing response to checkpoint inhibitors and should be taken into account in immune targeting trials in esophageal and gastric cancers.

## Data availability statement

The raw data supporting the conclusions of this article will be made available by the authors, without undue reservation.

## Ethics statement

The studies involving humans were approved by Medisch Etische Toetstingscommissie Amsterdam UMC. The studies were conducted in accordance with the local legislation and institutional requirements. The participants provided their written informed consent to participate in this study.

## Author contributions

TdG: Conceptualization, Data curation, Formal analysis, Investigation, Methodology, Project administration, Software, Validation, Writing – original draft, Writing – review & editing. MH: Conceptualization, Writing – original draft, Writing – review & editing, Data curation, Formal analysis, Investigation, Methodology, Project administration, Software, Validation. JS: Writing – original draft, Writing – review & editing, Data curation, Formal analysis, Investigation, Validation, Visualization. EB: Writing – original draft, Writing – review & editing, Investigation, Project administration, Validation. TF: Writing – original draft, Writing – review & editing. MvM: Investigation, Project administration, Writing – original draft, Writing – review & editing, Validation. RP: Investigation, Writing – original draft, Writing – review & editing. RG: Investigation, Writing – original draft, Writing – review & editing, Project administration. BD: Investigation, Project administration, Writing – original draft, Writing – review & editing. JS: Writing – original draft, Writing – review & editing, Data curation. JB: Writing – original draft, Writing – review & editing, Methodology. MvBH: Writing – original draft, Writing – review & editing, Investigation. VT: Writing – original draft, Writing – review & editing, Supervision. NvG: Writing – original draft, Writing – review & editing, Investigation. HvL: Conceptualization, Supervision, Writing – original draft, Writing – review & editing. TdG: Conceptualization, Supervision, Writing – original draft, Writing – review & editing. SD: Funding acquisition, Resources, Supervision, Writing – original draft, Writing – review & editing, Conceptualization.
