# Supplementary figures and images for: Phenotypic immune characterization of gastric and esophageal adenocarcinomas reveals profound immune suppression in esophageal tumor locations
Source: Front Immunol. 2024 Apr 4;15:1372272. doi: 10.3389/fimmu.2024.1372272 (PMC11024289; doi:10.3389/fimmu.2024.1372272)

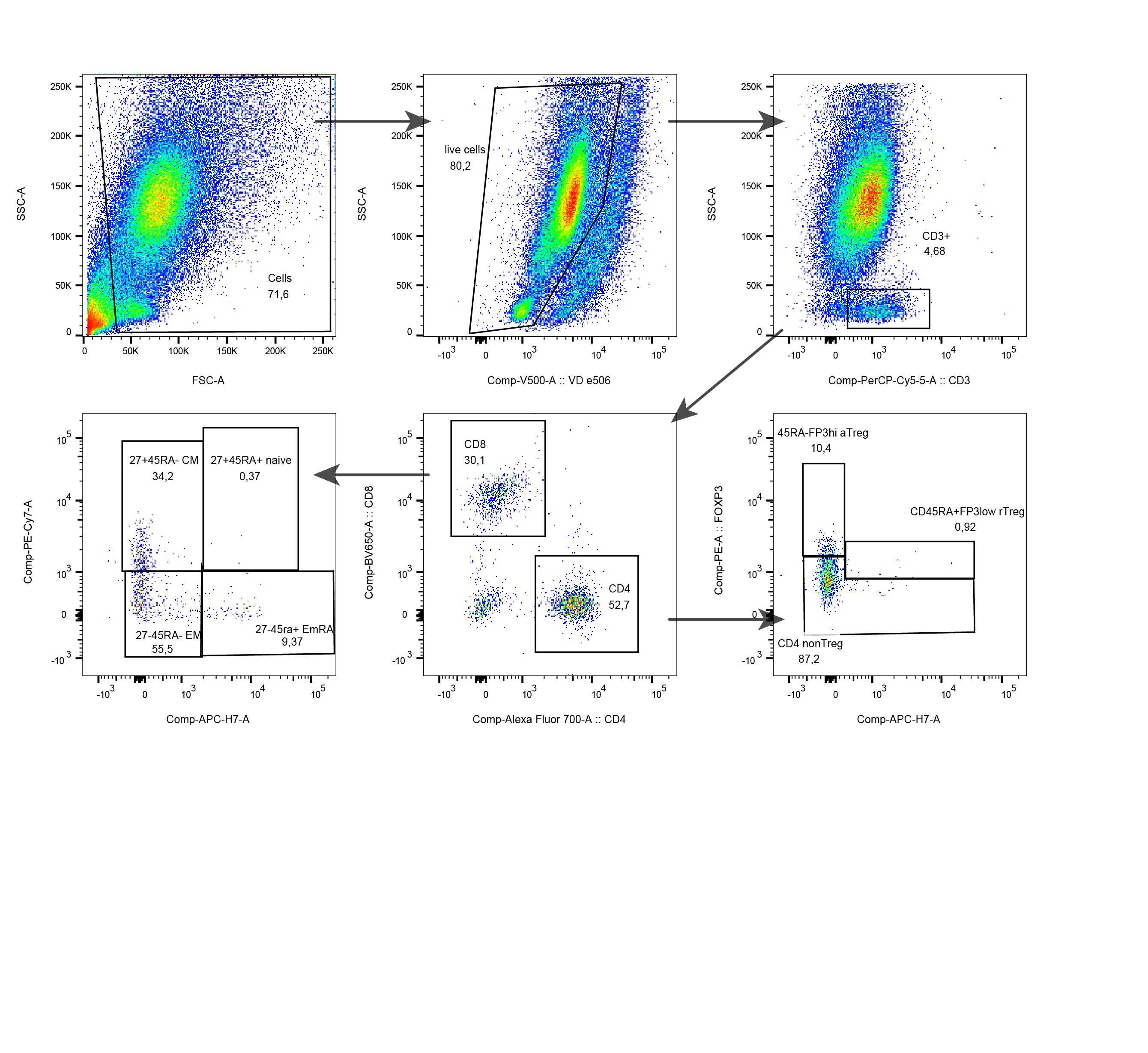

Supplement: Supplementary file 1 [file Image_1.jpeg]

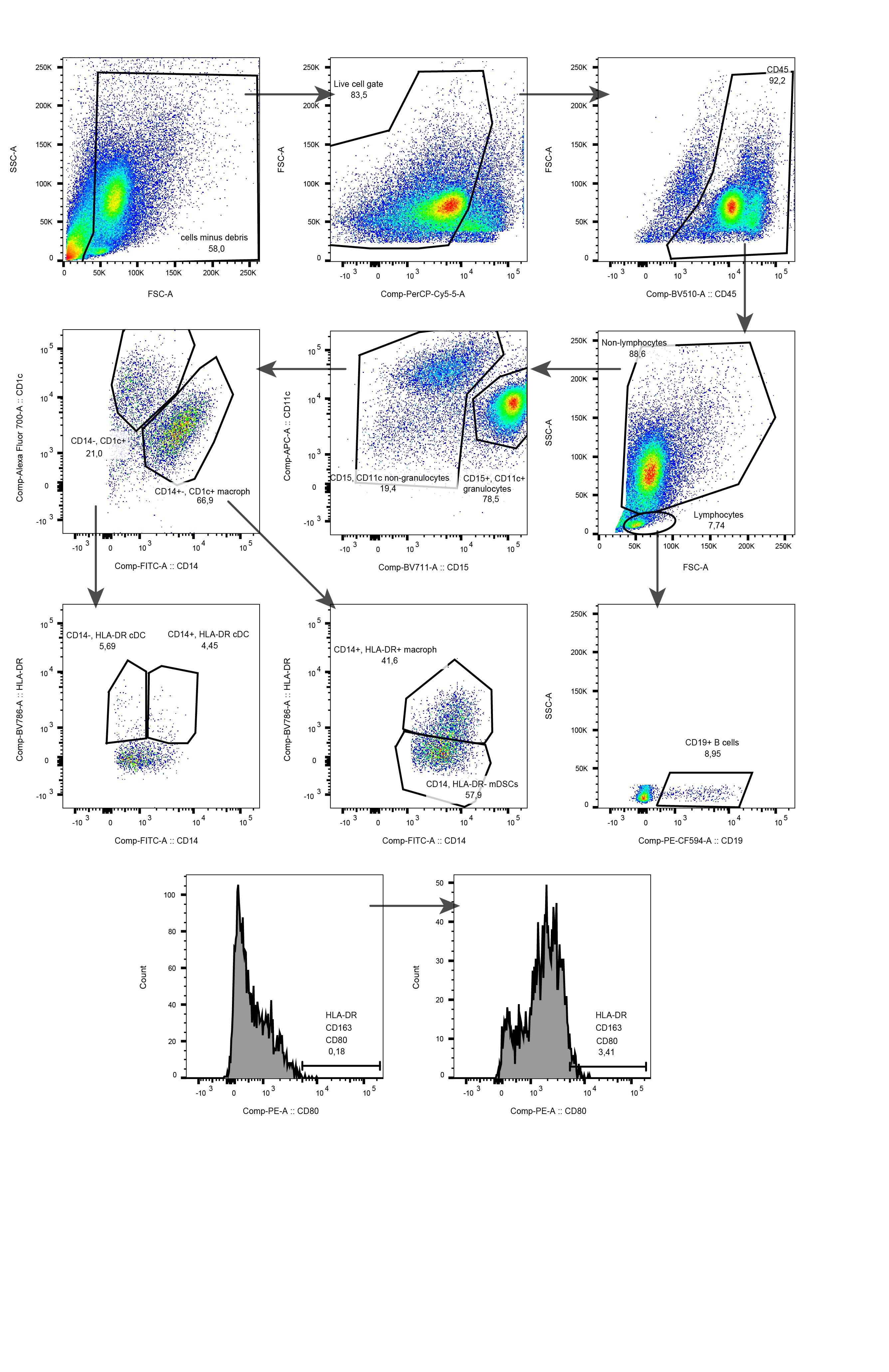

Supplement: Supplementary file 2 [file Image_2.jpeg]
